# Prevalence, aetiology and antibiotic sensitivity profile of asymptomatic bacteriuria isolates from pregnant women in selected antenatal clinic from Nairobi, Kenya

**DOI:** 10.11604/pamj.2017.26.41.10975

**Published:** 2017-01-30

**Authors:** Adelaide Ogutu Ayoyi, Gideon Kikuvi, Christine Bii, Samuel Kariuki

**Affiliations:** 1 College of Health Sciences Jomo Kenyatta University of Agriculture and Technology, Kenya; 2 College of Public Health Jomo Kenyatta University Agriculture and Technology, Centre for Microbiology Research Kenya Medical Research Institute, Kenya

**Keywords:** Asymptomatic bacteriuria, pregnant women, Kenya

## Abstract

**Introduction:**

Asymptomatic bacteriuria (ASB) is the presence of bacteria in urine without apparent symptoms of urinary tract infections. The importance of asymptomatic bacteriuria lies in the insight it provides into symptomatic infections. To determine prevalence, bacterial isolates and Antibiotic Sensitivity Profile of asymptomatic bacterial urinary tract infection in pregnant women in selected clinics in Nairobi.

**Methods:**

This was a cross-sectional study involving women attending antenatal clinic at selected clinics of Nairobi County. The women who met the inclusion criteria were included in the study. The midstream urine samples of these women were subjected to microscopy, culture and sensitivity.

**Results:**

A total of 1020 of women on their first antenatal clinic visit participated in the study; 219 of them had ASB, giving a prevalence of 21.5 % at 95% confidence level. *Escherichia coli* were the common organism isolated at 38.8%. The majority of the organisms were sensitive to imipenem and gentamycin.

**Conclusion:**

There is a high prevalence of ASB among pregnant women included in the study from the Nairobi county clinics. Therefore, routine ASB screening of pregnant women is recommended among the women attending antennal clinics in Nairobi county clinics.

## Introduction

Bacteriuria is the presence of bacteria in the urine. Bacteriuria is said to be significant in the presence of > 10^5^colony forming units (CFU)/ml or more of pure isolates [1, 2]. The danger with bacteriuria is that it does not always present with symptoms [2]. However asymptomatic bacteriuria during pregnancy if left untreated, may lead to acute pyelonephritis, preterm labour, low birth weight foetus [3, 4]. Profound physiologic and anatomic changes in the urinary tract during pregnancy contribute to the increased risk for infection [1, 5]. *E. coli* is a significant causative agent given it accounts for 80% to 90% of UTIs [6]. Most UTIs are caused by bacteria and are treated by antibiotics. However there is increased development of microbial resistance to these antibiotics [7, 8]. Drug resistance is an important phenomenon associated with increased treatment costs. A high proportion of the women who attend Nairobi county council clinics belong to the low socioeconomic class, have high parity, and reside in informal settlement setup. These have been demonstrated as risk factors for ASB by studies done earlier [9, 10]. It was because of this that study was done in the selected clinics. This study aimed to determine the profile, prevalence, microbiological isolates, and risk factors of ASB among pregnant women in Riruta, Kangemi and Kahawa Soweto area. The findings from this study may encourage the commencement of routine urine culture, and sensitivity at clinics in the study centres. It may also help determine the types of antibiotics to be used on pregnant women who have ASB.

## Methods

### Study area

Nairobi, Kenya’s capital city is a typical sub-Saharan Africa (SSA) urban centre characterized by population explosion with a current population of about 3.4 million in 2010 [11]. Majority of residents estimated at 60-70% of her population live in informal settlements [12]. The Nairobi county health clinics are distributed within the county and some cater for specialized needs like dental, skin and infectious conditions. Among the health clinics there are those that provide services from antennal to normal delivery. These are the ones we selected according their location in the informal settlement areas which are known risk factor for asymptomatic bacteriuria. The clinics run antenatal clinics on daily basis and all complicated cases are referred to specialized county hospital within Nairobi.

### Study design

This was a cross-sectional study of pregnant women on their first antenatal clinic of the Nairobi county health centres. A questionnaire was used to obtain information from the study participants. The information obtained consisted of identification number, age, phone number, educational level, marital status, parity, gestational age, and human immune virus status. The inclusion criteria involved the first visits of apparently healthy pregnant women attending clinic for first the visit those who gave their informed consent to participate in the study. However, the women excluded from the study were those who had features of urinary tract infection, fever, had taken antibiotics within 2 weeks of the study, had medical chronic conditions (HIV) retroviral disease, and those who declined to consent despite adequate counselling. All pregnant women at the antenatal clinic, who met the inclusion criteria, were counselled on how to collect midstream urine. This involved initial instructions by the female attending trained nurses and laboratory technicians. The laboratory technicians supervised the urine-sample collection. The first part of the urine was voided, and approximately 10-15 mL of midstream urine was collected in a sterile universal bottle, that had been correctly labelled and given to the patients. The urine samples in the sterile universal bottles were taken to the laboratory for processing within 1 hour. These samples were subjected to culture, and sensitivity according to standard. Microscopy was done at the clinic involved centrifugation of approximately 10 mL of urine sample in a tube at 1,500 rpm. The sediments were poured on a clean slide and observed under a microscope for casts, pus cells, and yeast cells. The remaining urine was sent to processing laboratory for culture Samples were cultured on air dried plates of Cysteine lactose electrolyte deficient agar (CLED) using a calibrated loop delivering 0.002ml of urine. Plates were incubated aerobically at 37°C overnight. Colony counts yielding bacterial growth of 10^5^ organism/ml or more of pure isolates were deemed significant. Contaminated urine usually has less than 104organism/ml and often contains more than one bacteria species [13]. Suspected bacterial species were characterised by colonial morphology, gram stain followed by microscopic examination, motility test and biochemical tests. Isolates were identified to species level using standard methods according to Clinical and Laboratory Standard Institute Guidelines [14]. Following identification, the isolated microbes were subjected to antibiotic sensitivity testing using the disk diffusion technique. Multidisc (Oxoid; Thermo Fisher Scientific, Waltham, MA, USA) was used and interpreted in accordance with the zone-size interpretative chart. Kirby-Bauer disk diffusion test was used to perform antimicrobial susceptibility testing for all the isolates as recommended by Clinical and Laboratory Standard Institute [14].

### Data analysis

Data analysis was descriptive and multinomial logistic regression analysis was done at 95% confidence level using SPSS version 23.0 (IBM SPSS statistics Inc., Chicago, IL, USA). A P-value of less than or equal to 0.05 was considered statistically significant.

### Sample size

The formula used for sample size calculation was n=z^2^ (p (1-p))/e^2^ [15]. This study used an estimate of the proportion of population falling into the group of interest at 50%. The prevalence of asymptomatic bacteriuria in pregnancy in low socio-economic population and with specific inclusion criteria is unknown in Kenya. This gave the minimum sample size at 384 however due large patient numbers that are attended at the clinics of interest the sample size was increased for better representation.

### Ethical clearance

Ethical clearance for this study was obtained from the KEMRI Ethics Clearance Committee.

## Results

A total of 1020 women visiting the antenatal clinic for first visit who met the inclusion criteria participated in the study. The age of the women included in this study ranged between 16 to 45years with a mean of 24.3±4.6 years (P=0.053). A total 219 of the participants had ASB, giving a prevalence of 21.5%(95 CI range 19.1-23.9%). Table 1 shows the prevalence of asymptomatic bacteriuria from cultured urine. Table 2 shows the frequency of distribution of the bacterial isolates from positive urine cultures. The commonest bacteria isolated was *Escherichia coli* 38.8%, this was followed by Staphylococcus aureus at 29.7%. Then *coagulase negative staphylococcus* (13.2%), *Klebsiella* (7.8%), *Pseudomonas spp* (2.7%), *Protues spp* (2.7%), *Citrobacter* (2.3%), *Enterococcus* (1.9%) and *Enterobacter* (0.9%) respectively. Table 3 shows that age, parity, education and gestational age of the participants did not have any statistical significant association with presence of bacteriuria (P= 0.05) Multinomial logistic regression analysis of demography characteristics as associated risk factors revealed that age of the pregnant women (taking age range 15–21 years as a reference, 22–31 years; P= 0.993, OR= 1.518E-7, CI= 0), gestational age (taking last trimester as a reference, first trimester; P = 0.836, OR=1.086, CI=0.497,2.371, second trimester; P=0.554, OR=1.176, CI= 0.688, 2.011), parity (taking 2-3 as a reference: 0-1; P= 0.637, OR= 1.108, CI= 0.725, 1.693, ≥ 4; P= 0.650 OR= 0.800, CI= 0.305, 2.099), educational status (taking higher education as a reference, Non; P=0.385, OR=2.705, CI=0.286, 25.554, Primary; P=0.313, OR=3.140, CI=0.340, 28.992, Secondary; P=0.363, OR=2.822, CI=0.301, 26.446) were not significantly associated with ASB (Table 3). The resistance and susceptibility pattern of gram positive and gram negative isolates from positive asymptomatic bacteriuria is shown in Table 4 and Table 5. All bacterial isolates had high resistance to ampicillin ranging from 83.3% to 100%. Imipenem had the lowest resistance to all isolates ranging from 0% to 10.2% and most isolates were susceptible to imipenem ranging from 89.7% to 100%. *E. coli* isolates from this study had resistance to most antibiotics tested except for imipenem. The resistance ranged from 11.6% for gentamycin and to 90.6% for ampicillin. This study found 18.8% resistance to ciprofloxacin by the *E.coli* isolates.

**Table 1:** Prevalence of asymptomatic bacteriuria

Bacteriuria	Frequency	Percent	Range at 95%CI
Positive	219	21.5%	19.1%-23.9%
Insignificant	287	28.1%	25.5%-31.1%
Sterile	321	31.5%	28.6%-34.4%
Contamination	193	18.9%	16.6%-21.4%
Total	1020	100.0%	100.0%

**Table 2:** Distribution of bacteria isolated from positive sample

Type of bacteria isolates	Frequency	Percent
*Escherichia coli*	85	38.8%
*Staphylococcus aureus*	65	29.7%
*non coagulase staphylococcus*	29	13.2%
*Klebsialla spp*	17	7.8%
*Pseudomonas spp*	6	2.7%
*Protues spp*	6	2.7%
*citrobacter*	5	2.3%
*Enterococcus*	4	1.8%
*Enterobacter*	2	0.9%
Total	219	100.0%

**Table 3:** Logistic regression analysis of association of demographic characteristics with asymptomatic bacteriuria

Demographic Factors	Asymptomatic Bacteriuria	Sterile	insignificant	Contaminated	OR	P value	(95%CI)
**Age of patient (years)**							
15-21	73	110	80	67			
22-31	127	188	185	104	-	0.993	0.000
32-45	18	23	22	22			
˃45	1	0	0	0			
**Marital status**							
Married	160	242	140	140	0.873	0.506	0.585,1.303
Single	59	79	60	53			
**Parity**							
0-1	152	218	172	120	1.108	0.637	0.725,1.693
2-3	59	89	104	66	-	-	-
˃4	8	14	11	7	0.800	0.650	0.305,2.099
**Education level**							
Primary	82	112	105	72	3.140	0.313	0.340,28.992
Secondary	45	66	68	42	2.822	0.363	0.301,26.446
College	57	85	79	47	2.851	0.357	0.307,26.458
University	1	4	0	1	-	-	-
Non	34	54	35	31	2.705	0.385	0.286,25.554
**Trimester (weeks)**							
0-14	18	28	30	19	1.086	0.836	0.497,2.371
15-28	176	251	219	145	1.176	0.554	0.688,2.011
29-40	25	42	38	29	-	-	-
P ≤ 0.05							

**Table 4:** Antibiotic sensitivity pattern of gram negative isolates in asymptomatic bacteriuria

Antibiotics		E. coli	Proteus Spp	Klebsialla Spp	Pseudomonas Spp	Enterobacter Spp	Citrobacter spp
		***n=85***	***n=6***	***n=17***	***n=6***	***n=2***	***n=5***
Ampicillin	R S	77(90.6%) 2(2.4%)	6(100%) 0(0%)	15(88.2%) 2(11.8%)	5(83.3%) 1(16.7%)	2(100%) 0(0%)	5(100%) 0(0%)
Tetracycline	R S	51(60.0%) 26(30.6%)	6(100%) 0(0%)	12(70.6%) 5(29.4%)	6(100%) 0(0%)	2(100%) 0(0%)	5(100%) 0(0%)
Chloramphenicol	R S	17(17.0%) 60(70.6%)	4(66.6%) 1(16.7%)	5(29.4%) 11(64.7%)	0(0%) 6(100%)	0(0%) 2(100%)	2(40.0%) 3(60.0%)
Cotrimoxazole	R S	56(65.9%) 26(30.6%)	4(66.7%) 2(33.3%)	11(64.7%) 6(35.3%)	3(50.0%) 3(50.0%)	2(100%) 0(0%)	4(80.0%) 1(20.0%)
Ciprofloxacin	R S	16(18.8%) 63(74.1%)	6(100%) 0(0%)	4(23.5%) 13(76.5%)	0(0%) 6(100%)	1(50.0%) 1(50.0%)	0(0%) 5(100%)
Nalidixic acid	R S	36(42.4%) 47(55.3%)	1(16.7%) 5(83.3%)	4(23.5%) 12(70.6%)	0(0%) 4(66.7%)	1(50.0%) 1(50.0%)	1(20.0%) 2(40.0%)
Ceftazidime	R S	16(18.8%) 61(71.8%)	2(33.3%) 3(50.0%)	8(47.1%) 7(41.2%)	3(50.0%) 3(50.0%)	1(50.0%) 1(50.0%)	0(0%) 3(60.0%)
Gentamycin	R S	10(11.8%) 71(83.5%)	0(0%) 6(100%)	3(17.6%) 13(76.5%)	0(0%) 6(100%)	0(0%) 2(100%)	0(0%) 2(100%)
Amoxy/clavu	R S	49(57.6%) 19(22.4%)	3(50.0%) 0(0%)	9(52.9%) 4(23.5%)	3(50.0%) 0(0%)	1(50.0%) 1(50.0%)	4(80.0%) 1(20.0%)
Cefotaxime	R S	63(74.1%) 2(2.4%)	3(50.0%) 1(16.7%)	14(82.4%) 0(0%)	2(33.3%) 0(0%)	2(100%) 0(0%)	4(80.0%) 0(0%)
Imipenem	R S	0(0%) 85(100%)	0(0%) 6(100%)	1(5.9%) 16(94.1%)	0(0%) 6(100%)	0(0%) 2(100%)	0(0%) 5(100%)
Kanamycin	R S	66(77.6%) 14(16.5%)	0(0%) 6(100%)	5(29.4%) 10(58.8%)	2(33.3%) 4(66.7%)	1(50.0%) 1(50.0%)	2(40.0%) 3(60.0%)

**Table 5:** Antibiotic sensitivity pattern of gram positive isolatesin asymptomatic bacteriuria

Antibiotics		Staph aureus	Non coagulase staph	Enterococcus
		n=65	n=29	n=4
Ampicillin	R S	60(92.3%) 2(3.1%)	22(75.9%) 4(13.8%)	2(50.0%) 0(0%)
Tetracycline	R S	33(50.7%) 28(43.1%)	14(48.3%) 10(34.5%)	1(25.0%) 2(50.0%)
Chloramphenicol	R S	21(32.3%) 42(64.6%)	6(20.7%) 23(79.3%)	0(0%) 2(100%)
Cotrimoxazole	R S	37(56.9%) 27(41.5%)	22(75.9%) 5(17.2%)	3(75.0%) 1(25.0%)
Ciprofloxacin	R S	24(36.9%) 41(63.1%)	12(41.4%) 17(58.6%)	2(50.0%) 2(50.0%)
Ceftazidime	R S	41(63.1%) 19(29.2%)	15(51.7%) 11(37.9%)	3(75.0%) 0(0%)
Gentamycin	R S	17(26.2%) 48(73.8%)	10(34.5%) 19(65.5%)	2(50.0%) 1(25.0%)
Amoxy/clavua	R S	37(56.9%) 28(43.1%)	12(41.4%) 17(58.6%)	2(50.0%) 2(50.0%)
Cefotaxime	R S	61(93.8%) 0(0%)	25(86.2%) 0(0%)	3(75.0%) 0(0%)
Imipenem	R S	4(6.2%) 60(92.3%)	3(10.3%) 26(89.7%)	0(0%) 4(100%)
Kanamycin	R S	16(24.6%) 45(69.2%)	6(20.7%) 20(67.0%)	3(75.0%) 0(0%)

## Discussion

The current study reported a prevalence of ASB among women attending antenatal clinic at selected clinic in Nairobi at 21.5%. The prevalence of asymptomatic bacteriuria is not uncommon during pregnancy. However the importance of asymptomatic bacteriuria lies in the insight it provides into symptomatic infections [16]. Asymptomatic bacteriuria may exist for short term in non-pregnant women but rarely resolves spontaneously during pregnancy [17]. The prevalence of asymptomatic bacteriuria does not change during pregnancy but there is change in pathogenesis, which keeps mother and baby at risk of complications due to urinary tract infection [1]. The prevalence proportion reported in this study ranged between 19.1% to 23.9% and agreed with studies done in Tanzania, Ibadan and sagamu which reported prevalence of 21.0%,21.0% and 23.9% respectively [18, 19]. This was however higher than a study done in Kano that found prevalence at 10.2% [20]. There are however other studies which have reported much high prevalence proportions of 78.7% and 86.6% at Abakaliki and Benin, respectively [21, 22]. The current study prevalence also agreed with a study done in Kenya which reported a prevalence of 26.7% among pregnant women with symptomatic UTI [23]. In pregnancy there is a great relation between asymptomatic UTI and symptomatic UTI. It has been established that untreated asymptomatic UTI develop into symptomatic UTI [1, 24]. This explains why the current study prevalence agreed with the earlier study’s prevalence. Although the earlier study looked at symptomatic UTI and current study looked at asymptomatic UTI they both used similar subjects pregnant women and both studies were done in Nairobi. Prevalence of asymptomatic bacteriuria in pregnant women in the world is known to vary from geographical region to region even within the same country. Studies done in Iran demonstrated different prevalence proportons in the same country but different localities at Semnan public health centres was 3.3% while at Tabriz centres was 6.1% [25, 26]. However the prevalence in the current study was found be within the range of earlier studies done in the same locality and a different locality at Nairobi was 26.7% and at Gucha was 19.7% respectively [23, 27]. Although inclusion criteria for the earlier studies were quite different from this study. There are many risk factors that are thought to predispose pregnant women to asymptomatic bacteriuria. It has been demonstrated that the asymptomatic urinary tract infection incidences increase with number of children, recurrent urinary tract infection and was also noted that most of the infected subjects are in their first and early second trimester [28].

However age, parity, education level, and gestational age of the participants did not have any statistical significant influence on ASB in this study. The age distribution of the participants in this study appeared not to have any significant association on ASB (P = 0.05). Parity, not having an influence on ASB, in this study is similar to previous reports in Ibadan, Nigeria, Ghana and Iran [29–31]. However this did not agreed with another study where ASB in pregnancy was associated with increasing parity [32]. The prevalence of ASB across the trimesters in this study showed no consistent pattern of influence. It did not tally either with observations by Awonuga found increasing prevalence with gestational age, or Nnatu who found decreasing prevalence with duration of pregnancy [29, 30]. Trimester not having a significant effect on ASB in this study is similar to a previous report in Benin [33]. The age distribution of the participants in this study appeared not to have any statistical significant association on prevalence of ASB. This is agreed with a study done in Abakaliki Nigeria [29]. However other studies have reported advancing maternal age having significant association on prevalence of ASB [34]. The lack of significant effect of age on prevalence ASB in this study may have been due the factor the 91.6% of the women included in this study were between the ages of 15 - 31 years. Those between 32-45 years were 8.6% therefore statistically the number of women of advanced age was low. Educational level attained may be an indicator of the socioeconomic status of the women. Lower levels of education and low socioeconomic status have been related to higher prevalence of ASB in many studies and reports [35]. This is because education improves the attitudes and beliefs of women. However education level of the participants in this study did not have any significant association with ASB, which agreed a study done by Onu [29].

Other earlier studies have reported education level having a significant association with ASB [35]. This could have been because only 15% of the study subjects had no basic education background while 85% had either attained a primary, secondary, college or university education levels. The bacteria isolated from urine cultures were *Escherichia coli* (38.8%) *Staphylococcus aureus* was (29.7%), Non coagulase *Staphylococcus* was (13.2%), *Klebsiella spp* was (7.8%), Pseudomonas spp was (2.7%), *Protues spp* was (2.7%), *Citrobacter spp*was (2.3%), *Enterococcus spp* was (1.9%) and Enterobacter spp was (0.9%) respectively. *Escherichia coli* was the most common uropathogen isolated in this study (38.8%). This study agreed with a study by Ankur in India which found organism isolated as follows Escherichia coli (39.47%) followed by *Staphylococcus aureus* (23.68%), *Staphylococcus saprophytic* us (10.52%), Enterococcus spp (7.89%), Klebsiella spp (5.26%), Proteus spp (5.26%) and *Pseudomonas aeruginosa* (2.63%) [36]. This was also in agreement with findings made by Mokube in Cameroon and Akoachere in Buea [37, 38]. Another study conducted by Amadi and colleagues demonstrated Staphylococcus aureus as the most prevalent uropathogen. However in this study found staphylococcus aureus as the second most common at 29.7%. Other studies have demonstrated different bacterial species as dominant uropathogen like *Klebsiella* [38]. In this study the Gram negative bacteria were more prevalent at (55.3%) than Gram positive bacteria which were (44.71%). This was in agreement with a study done in Tanzania which found Gram negative bacteria were more prevalent at (61.9%) and Gram positive bacteria at (38.1%) [18] and Demilie also observed similar patterns in bacterial isolates [39]. *E. coli* was major pathogen isolated from the urine cultures and accounted for one-third of the positive cultures with significant bacteriuria. *E. coli* is considered major uropathogen due to a number of virulence factors specific for colonization and invasion of the urinary epithelium [40] (Sheffield & Cunningham, 2005).

In overall gram negative bacteria have a unique structure which assists in attachment to the uro-epithelium and prevent pathogens from being washed away by urine. The same characteristics allow for growth and tissue invasion resulting in invasive infection and pyelonephritis during pregnancy [41]. While the range of etiological agents causing asymptomatic and symptomatic UTI in pregnant women is relatively constant, the susceptibility profile is different in different geographical locations. All bacterial isolates had high resistance to ampicillin ranging from 83.3% to 100%. Imipenem had the lowest resistance to all isolates ranging from 0% to 10.2% and most isolates were susceptible to Imipenem ranging from 89.7% to 100%. *E.coli* isolates from this study had resistance to most antibiotics tested except for Imipenem. The resistance ranged from 11.6% for gentamycin and to 90.6% for ampicillin. The current study showed high level of resistance to first line antimicrobial drugs such as cotrimoxazole. These findings agree with findings from previous studies [18, 42]. The high resistance to cotrimoxazole observed in the current study call for the need to strengthen surveillance to identify changes in sensitivity pattern among urinary tract isolates. The current study also showed high level of cefotaxime resistance in overall it was 90.8%. The isolates with the highest resistance were *E. coli* (74.1%) and *Staphylococcus aureus* (93.8%). Cefotaxime is second line antimicrobial agent in the third generation of cephalosporins. A possible explanation for the resistance found might be the presence of mobile resistant factors in these strains. There were 17(14.0%) of the gram negative isolates which were resistant to Cefotaxime, Ceftazidime and amoxicillin-cluvulanic acid. An earlier study done in Kenya reported similar resistance pattern in uropathogen *E. coli* in Kenya [43]. This could be an indication of co-selection of the genes conferring resistance to these antibiotics. The emergence of ESBLs in community-acquired infections has been reported in earlier studies [42].

Resistance to ciprofloxacin was at 18.8% for the *E. coli* isolates although relatively low, this trend needs to be watched closely. Since Fluoroquinolones are thought be still the most effective antibiotic agents against *E. coli*infection [44]. This is only true to some localities otherwise resistance to Fluoroquinolones has been increasing in other localities. There is increased resistance to Fluoroquinolones such as ciprofloxacin and levofloxacin. Kenya has reported development of resistance to Fluoroquinolones and extended-spectrum beta-lactams in uropathogenic *E. coli* [43]. This study found 18.8% resistance to ciprofloxacin by the *E. coli* isolates. In this study there were some isolates which showed resistance to imipenem one (1) Klebsiella spp, four (4) of staphylococcus aureus and three (3) coagulase negative staphylococcus spp. A study done earlier in Kenya in a hospital setup found one (1) isolate was found to be resistant to imipenem [45]. While this class of drugs remains useful for treating serious infections due to multidrug resistant enteric bacteria in our set up, the few isolates resistant to carbapenems are clinically relevant and suggest that this pattern of resistance should be monitored closely. Antibiotic use drives the evolution of resistance [46]. Resistant bacteria spread by selection pressure when antibiotics are used, drug susceptible bacteria are destroyed leaving resistant ones to proliferate. Antibiotic resistance management is an attempt to slow the spread of resistance by the judicious use of antibiotics. However overuse of antibiotics clearly drives the evolution of resistance [47, 48]. Epidemiological studies have demonstrated a direct relationship between antibiotic consumption and the emergence and dissemination of resistant bacteria strains [46]. In the U.S., the sheer number of antibiotics prescribed indicates that a lot of work must be done to reduce the use of these medications [49]. Despite warnings regarding overuse, antibiotics are overprescribed worldwide [47].

This study limitation was the health clinics based design catchment area, which may not be a true reflection of what is happening in Nairobi. However, it was strengthened by the random sampling of the health centres which was from three different sub counties.

## Conclusion

In conclusion, there is a high prevalence of ASB among pregnant women in Nairobi. Demographic characteristics and educational level did not significantly influence the risk of ASB in this study population. *E. coli* and *S. aureus* were the dominant bacterial isolates. Low to moderately high level of resistance against first line drugs and high level of resistance against third generation cephalosporin was observed. It is recommended to monitor the levels of resistance for Fluoroquinolones and cefotaxime and screen for Extended Spectrum Beta Lactamase production among cefotaxime resistant *E. coli* and *Klebsiella.*

### What is known about this topic

- Prevalence of asymptomatic bacteriuria in pregnant women with HIV/AIDS was demonstrated at 19.7% in Kenya;
- The most common uropathogen was Escherichia coli;
- A high rate of resistance to tetracycline, cotrimoxazole and ampicillin has been reported.

### What this study adds

- Prevalence of asymptomatic bacteriuria in pregnant women without HIV/AIDS was found at 21.5% in Kenya in this study;
- High resistance against third generation cephalosporin was observed in this study and one isolate was resistant to Imipenem;
- The most common uropathogen was *Escherichia coli*.
